# SARS-CoV-2: Pathogenesis, Molecular Targets and Experimental Models

**DOI:** 10.3389/fphar.2021.638334

**Published:** 2021-04-22

**Authors:** G. Kanimozhi, B. Pradhapsingh, Charan Singh Pawar, Haseeb A. Khan, Salman H. Alrokayan, N. Rajendra Prasad

**Affiliations:** ^1^ Department of Biochemistry, Dharmapuram Gnanambigai Government Arts College for Women, Mayiladuthurai, India; ^2^ Department of Biochemistry, College of Science, King Saud University, Riyadh, Saudi Arabia; ^3^ Department of Biochemistry and Biotechnology, Annamalai University, Annamalainagar, India

**Keywords:** COVID-19, molecular targets, drug discovery, pathogenesis, SARS-CoV-2

## Abstract

The severe acute respiratory syndrome coronavirus 2 (SARS-CoV-2) is a recent pandemic outbreak threatening human beings worldwide. This novel coronavirus disease-19 (COVID-19) infection causes severe morbidity and mortality and rapidly spreading across the countries. Therefore, there is an urgent need for basic fundamental research to understand the pathogenesis and druggable molecular targets of SARS-CoV-2. Recent sequencing data of the viral genome and X-ray crystallographic data of the viral proteins illustrate potential molecular targets that need to be investigated for structure-based drug design. Further, the SARS-CoV-2 viral pathogen isolated from clinical samples needs to be cultivated and titrated. All of these scenarios demand suitable laboratory experimental models. The experimental models should mimic the viral life cycle as it happens in the human lung epithelial cells. Recently, researchers employing primary human lung epithelial cells, intestinal epithelial cells, experimental cell lines like Vero cells, CaCo-2 cells, HEK-293, H1299, Calu-3 for understanding viral titer values. The human iPSC-derived lung organoids, small intestinal organoids, and blood vessel organoids increase interest among researchers to understand SARS-CoV-2 biology and treatment outcome. The SARS-CoV-2 enters the human lung epithelial cells using viral Spike (S1) protein and human angiotensin-converting enzyme 2 (ACE-2) receptor. The laboratory mouse show poor ACE-2 expression and thereby inefficient SARS-CoV-2 infection. Therefore, there was an urgent need to develop transgenic hACE-2 mouse models to understand antiviral agents’ therapeutic outcomes. This review highlighted the viral pathogenesis, potential druggable molecular targets, and suitable experimental models for basic fundamental research.

## Introduction

The coronavirus disease-19 (COVID-19) is a pandemic outbreak caused by a novel severe acute respiratory syndrome coronavirus-2 (SARS-CoV-2) (Rothan and Byrareddy, 2020). This ongoing infectious outbreak was first noticed in Wuhan, China, in December 2019. The incidence of SARS-CoV-2 infection has alarmingly increased worldwide. As of January 2021, about 100,869,345 new SARS-CoV-2 infected patients and 2,174,143 deaths have been reported across 188 countries by the Center for Systems Science and Engineering (CSSE) of Johns Hopkins University (John Hopkins University and Medicine, 2020). Globally, as of February 8, 2021, there have been 105,805,951 confirmed cases of COVID-19, including 2,312,278 deaths, were reported by WHO [WHO Coronavirus Disease (COVID-19) Dashboard, 2020]. Although this infectious disease’s recovery rate was high, it remains a significant health issue as it causes mortality in aged and immunocompromized patients (Bialek et al., 2020; Russell et al., 2020a; Sarkar and Chakrabarti, 2020). Researchers demand a need for the coordinated global development effort using a “big science” approach to combat this deadly disease (Berkley, 2020).

The COVID-19 infection exhibits inter-individual variability. It causes a wide range of severity, from the asymptomatic career to patients with multiple organ failure (Pereira et al., 2021). Studies illustrate the COVID-19 disease manifestation and progression has been correlated with the age, race, ethnicity, sex and the expression pattern of ACE-2, and immune regulation of the individuals (Benetti et al., 2020; Penna et al., 2020). Thus, understanding inter-individual variability enables a precision medicine approach against COVID-19 infection. Epidemiological studies and genome-wide association studies illustrate genetic variation has been linked with individual differences in susceptibility to COVID-19 infection (Gibson et al., 2020). The genetic variants in the expression *ACE*, *ACE2*, and *TMPRSS2* might directly impact COVID-19 disease. Allele frequencies and single nucleotide polymorphisms (SNPs) in different ethnic populations were postulated to be the reason for differences in the prevalence of COVID-19 infection among individuals (Asselta et al., 2020). Age-adjusted hospitalization and mortality rates of COVID-19 show that males were significantly affected than females due to the variation in the expression pattern of ACE-2 receptors (Penna et al., 2020). Recent studies illustrate that individuals with blood group A and blood group O show higher and lower susceptibility to COVID-19 infection, respectively (Zhao J. et al., 2020). Several studies were currently undergoing to identify the inter-individual variation to COVID-19 infection to enable high-risk patients for therapeutic intervention and vaccination.

## Treatments for COVID-19 and Emergency Use Authorizations (EUAs)

The clinicians employ numerous drugs for the treatment of SARS-CoV-2 infection considering the emergency of the disease. The US Food and Drug Administration (2020) have started Coronavirus Treatment Acceleration Program (CTAP) immediately after the COVID-19 outbreak (US Food and Drug Administration, 2020). More than 570 drug development programs in planning stages, 270 plus trials have been reviewed, and two treatments were currently authorized for emergency (Krause and Gruber, 2020). However, several therapies currently employed against SARS-CoV-2 infection were mainly supportive and used to treat infection symptoms. Antiviral drugs are proposed for the treatment of COVID-19 infection (Phadke and Saunik, 2020). Several antiviral drugs were repurposed to manage SARS-CoV-2 infection (Andrade et al., 2020; Serafin et al., 2020). WHO has supported remdesivir and lopinavir to treat COVID-19 infection (Won and Lee, 2020). Further, the FDA has authorized remdesivir, an inhibitor of viral RNA polymerases, to use during emergency conditions (EUA) in hospitalized patients (Eastman et al., 2020). Currently, remdesivir is the only medication approved by the FDA to treat coronavirus disease 2019 (COVID-19) (Al-Tannak et al., 2020; Saha et al., 2020a). Therefore, the remdesivir has been considered a “molecule of hope” for treating this disease. The approval was based on findings that hospitalized patients who got remdesivir recovered faster. Several pharmaceutical companies are currently conducting clinical trials to prove the efficiency of remdesivir for SARS-CoV-2 treatment (Goldman et al., 2020).

Several drugs were repurposed to prevent and treat SARS-CoV-2 infection (Akhtar et al., 2020; Rocha et al., 2020). Non-steroidal anti-inflammatory drugs (NSAIDs) such as cyclooxygenase (COX) inhibitors were most commonly employed for the management of SARS-CoV-2 infection (Kakodkar et al., 2020). The Indian Council of Medical Research has recommended hydroxychloroquine as a chemoprophylaxis drug for asymptomatic confirmed patients (Rathi et al., 2020). The National Health Commission of the People’s Republic of China has advocated the inclusion of chloroquine phosphate to treat COVID-19 patients (Gao J. et al., 2020). However, severe concerns were raised over NSAID usage as they were associated with severe adverse effects (FitzGerald, 2020). Acute organ failure, opportunistic infections, and acute respiratory distress syndrome (ARDS) are the major adverse events associated with NSAIDs (Russell et al., 2020b). Ivermectin, an anthelmintic drug, has also been considered a potential drug candidate for COVID-19 treatment (Sharun et al., 2020a). Rapamycin, an inhibitor of rapamycin, has been repurposed for attenuating proinflammatory cytokines attach during COVID-19 disease (Husain and Byrareddy, 2020). A drug repurposing study illustrates that the antioxidants like polyhydroxy-1,3,4-oxadiazole compounds such as CoViTris2020 and ChloViD2020 behave as protein blockers of SARS-CoV-2 molecular targets with significant higher potencies (Rabie, 2021).

Both innate and adaptive immune responses were activated during SARS-CoV-2 infection (Sami et al., 2021). It could be possible to prevent COVID-19 infection by modulating natural innate immunity (Schijns and Lavelle, 2020). SARS-CoV-2 infection significantly increased the antibody production in the affected individuals (Mathew et al., 2020). The SARS-CoV-2 viruses were sensed by immune cells such as macrophages, monocytes, and dendritic cells, which resulted in the production of proinflammatory cytokines, which subsequently damages the respiratory epithelial cells of the lungs. The anti-inflammatory corticosteroids have widely been used to treat SARS-CoV-2 infection. Corticosteroids such as dexamethasone were used to manage the inflammatory responses during SARS-CoV-2 infection (Singh et al., 2020). Dexamethasone treatment has been reported to reduce IL-8 and IP-10 concentrations immediately after administration (Zha et al., 2020). Dexamethasone has been used to combat cytokinemia induced by COVID-19 infection (Sharun et al., 2020b). The dexamethasone treatment (6 mg once daily for 10 days) even reduced the mortality rate in COVID-19 patients (Rizk et al., 2020). Several non-specific immunomodulators include interferons, angiotensin modulators, statins, azithromycin, clarithromycin, and ramatroban (prostaglandin D2 modulators), were also found to be effective against SARS-CoV-2 infection (Gasparyan et al., 2020). A low-molecular-weight heparin molecule was useful in dealing with COVID-19-related coagulopathy (Bartoli et al., 2021).

Immunotherapy strategies for SARS-CoV-2 are current developments against SARS-CoV-2 infection (Ura et al., 2021). Currently, researchers are involved in developing antibody-based immunotherapeutics using convalescent plasma to counteract SARS-CoV-2 infection (Sharun and Dhama, 2020). The convalescent serum with neutralizing antibodies from the recently recovered patients has been successfully employed to treat SARS-CoV-2 infection (Robbiani et al., 2020). The antibodies present in the convalescent sera can bind to the SARS-CoV-2 virus and enhance phagocytosis of the viral particle through complement activation and antibody-dependent cellular cytotoxicity (Rojas et al., 2020).

Tocilizumab targets inflammatory IL-6 has already been used in the context of severe Covid-19 infection (Roumier et al., 2020). Similar immunomodulatory drugs such as sarilumab are under investigation for the treatment against SARS-CV-2 mediated inflammatory responses (Ku et al., 2021; Roumier et al., 2020). There were several registered clinical trials on tocilizumab, sarilumab, and eculizumab against COVID-19 infection (Dhama et al., 2020). The results illustrate that tocilizumab was relatively effective and safe compared to the other immunomodulators (Tang et al., 2020). The FDA has recently issued a EUA for an antibody cocktail of basiliximab and imdevimab to adult patients infected with the SARS-CoV-2 virus (Weinreich et al., 2021). A phase 3 randomized clinical trial with the mRNA-1273 vaccine showed greater (94.1%) efficacy against Covid-19 illness (Baden et al., 2020; Ura et al., 2021).

The SARS-CoV-2 infection mainly involves the respiratory system within 14 days of incubation (WHO, 2020). Severe pneumonia resulted in SARS-CoV-2 infection leading to respiratory failure, which resulted in mortality (Tay et al., 2020). There is an evident lack of scientific evidence to understand the pathogenesis and molecular signaling associated with SARS-CoV-2 infection (Wu Y.-C. et al., 2020). Understanding the molecular pathogenesis of SARS-CoV-2 infection might lead to specific inhibitors against this deadly viral pathogen. Identification of preclinical drug targets using genomic, proteomic, and chemoinformatic studies might provide deep insights into the development of effective and specific antiviral therapeutics against SARS-CoV-2 infection (Li X. et al., 2020). After the pandemic outbreak, the Chinese researchers quickly sequenced the viral genome and publicly available (Chan et al., 2020; Lu R. et al., 2020). This genomic sequence helped the researchers develop useful diagnostic kits and conduct clinical trials to repurpose the existing antiviral agents against SARS-CoV-2 infection.

**FIGURE 1:**
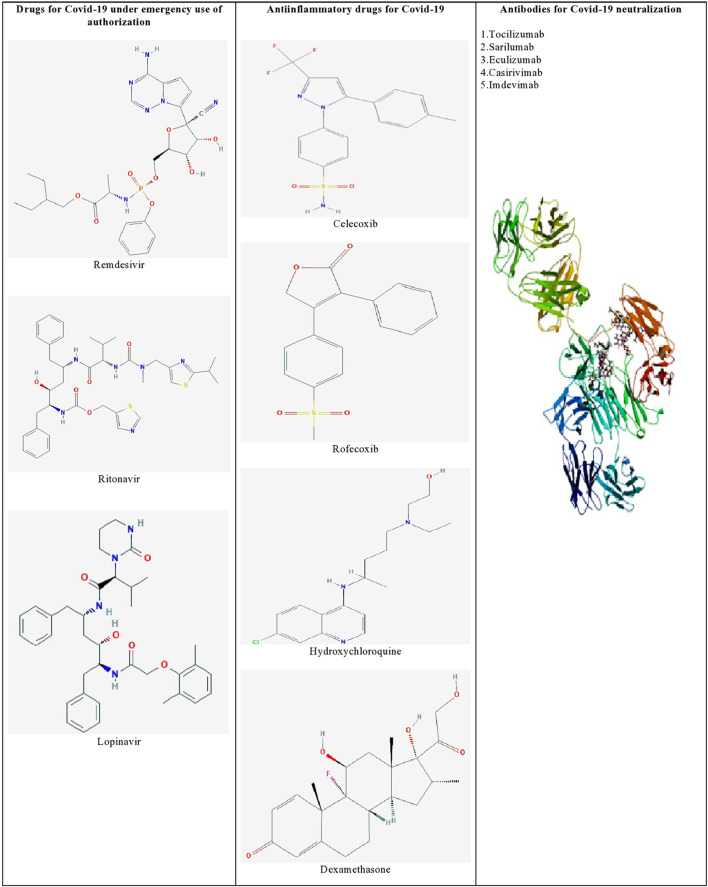
List of promising therapeutic candidates for SARS-CoV-2 infection. Remdesivir, ritonavir, and lopinavir were employed under emergency use of authorization. Anti-inflammatory drugs such as dexamethasone, hydroxychloroquine, rofecoxib were used to manage inflammatory responses during SARS-CoV-2 infection. Humanized monoclonal antibodies such as tocilizumab, sarilumab, eculizumab, casirivimab, and imdevimab were found to be effective against SARS-CoV-2 induced pneumonia.

Furthermore, identifying the novel molecular targets by understanding the viral-human protein interaction might lead to therapeutics against SARS-CoV-2 infection (Aziz et al., 2020). Gordon et al. (2020) identified 69 existing FDA-approved drugs by mapping the protein-protein interaction network of SARS-CoV-2 with human protein factors. Figure 1 shows the list of promising therapeutic candidates that were discussed in this review for SARS-CoV-2 infection.

## The Viral Genome and Its Proteins

The genome of SARS-CoV-2 was homologous to the genome of SARS-CoV that was responsible for the cause of severe acute respiratory syndrome (SARS) arisen during 2003 (Lu R. et al., 2020). The SARS-CoV-2 possesses a positive-sense single-stranded RNA as genetic material. The SARS-CoV-2 genome possesses 29,891 bp, which contains about 38% of GC content. This genome has 14 open reading frames (ORF) with a set of nine subgenomic mRNAs that possess a conserved leader sequence, nine regulatory sequences, and two terminal untranslated regions (UTR) that encode viral proteins necessary for the viral life cycle in the human lungs (Table 1).

**TABLE 1:** SARS-CoV-2 viral genome structure, types of viral proteins, and their function.

Genome	Number of ORFs	Type of protein encoded by the genome	Examples of enzymes/proteins encoded by the genome	The function of the enzymes/proteins
5′ end of the genome	Single ORF	Polyprotein that has been cleaved into 16 nonstructural proteins (NSP 1–6)	Replicase-transcriptase complex	Viral genome replication, RNA-dependent RNA polymerase, endonucleases, exonucelases
3′ end of the genome	13 ORFs	Structural proteins	Spike (S) protein, envelope (E) protein, membrane (M), and nucleocapsid (N) protein	Forms viral capsid; encapsulates viral genome; facilitates entry to human cells

**FIGURE 2:**
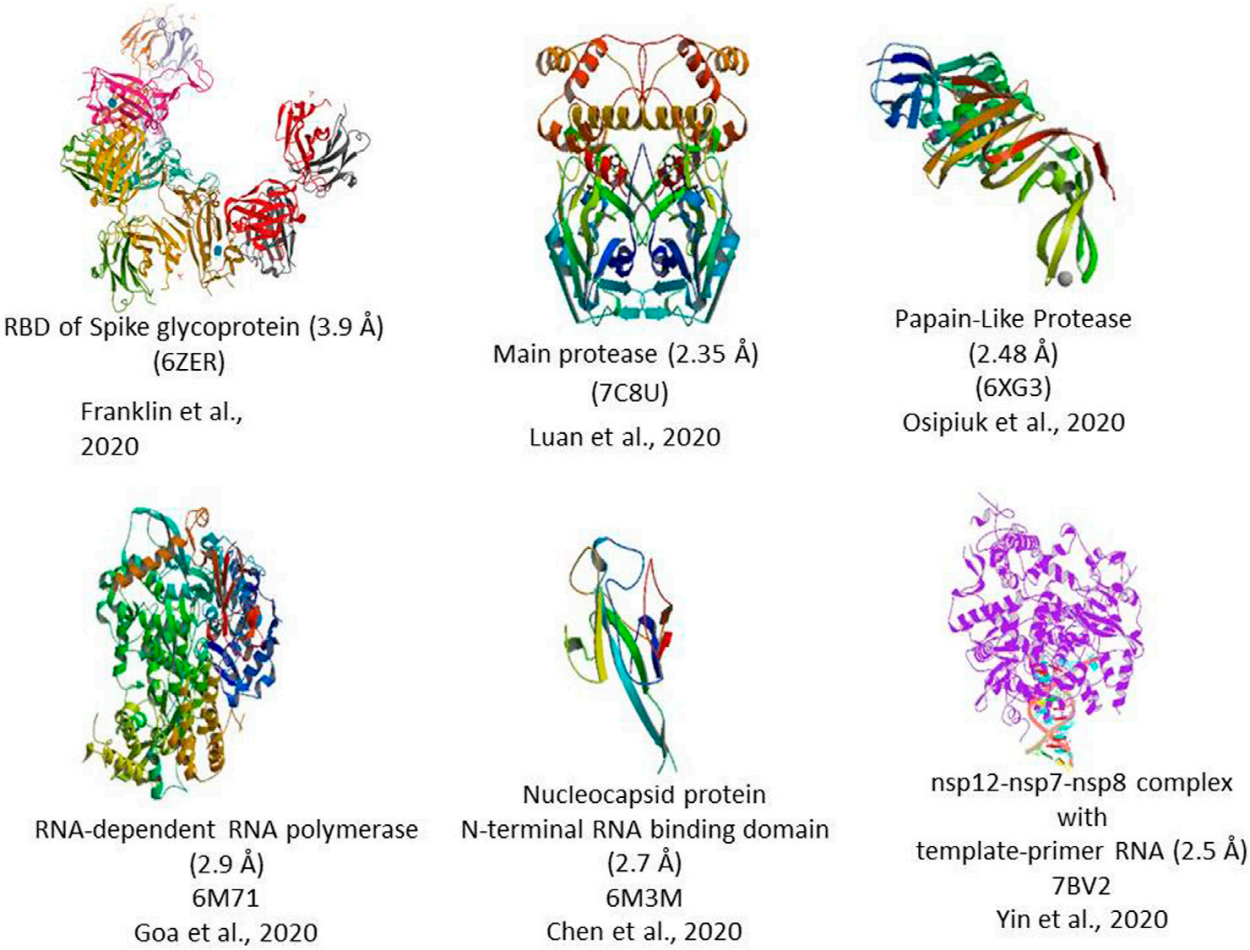
3D Crystal structure of prominent molecular targets of SARS-CoV-2. The structures were obtained from the Protein Data Bank (PDB).

The 5′ end of the viral genome contains a single ORF that codes for a polyprotein complex that automatically cleaved into 16 nonstructural proteins 1–16 (nsp 1–16). All these 16 nsp proteins are mainly replicase and transcriptase complex, including RNA-dependent RNA polymerase (NSP12), endonucleases, and exonucleases (Angeletti et al., 2020). The nsp also includes two viral cysteine proteases, namely, NSP3 (papain-like protease) and NSP5 (main protease), NSP13 (helicase), and other NSPs, which are likely involved in the transcription and replication of the virus. All the proteins formed from the 3′ end of the genome are involved in the viral genome replication. The 3′ end of the genome contains 13 ORFs, which code mainly structural proteins such as Spike (S), Envelope (E), Membrane (M), and Nucleocapsid (N). Structural proteins primarily perform two essential functions, such as the 1) formation of the viral capsid that encapsulates the viral genome and 2) facilitates the entry of the virus to the human cells through host receptors (Figure 2).

## Mechanism of SARS-CoV-2 Pathogenesis

**FIGURE 3:**
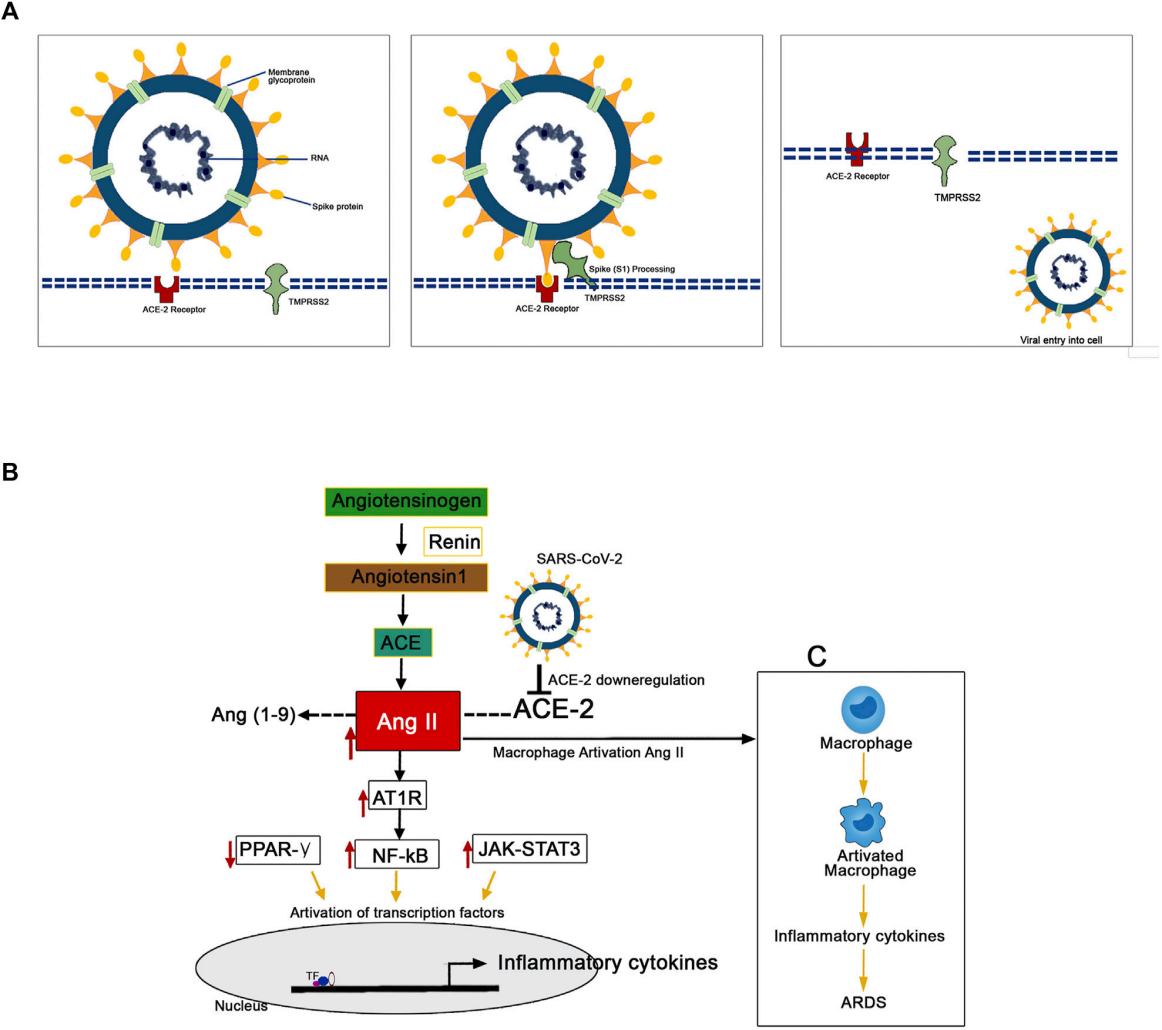
Mechanism of the pathogenesis of SARS-CoV-2 infection. **(A)** The TMPRSS2 process spike proteins for the binding with ACE-2 receptors present in the human epithelial cell membrane. **(B)** SARS-CoV-2 downregulates the expression of ACE-2 resulted in the upregulated expression pattern of Ang II. This Ang II binds with plasma membrane receptor AT1R and transduces signals to activate inflammatory transcription factors like NF-kB, STAT-3. These activated transcription factors are involved in the overexpression of several inflammatory. **(C)** The Ang II/AT1R interaction activates macrophages to produce excessive inflammatory cytokines that resulted in a “cytokine storm.”

The mechanism of pathology and the pathogenesis of SARS-CoV-2 infection has now been clearly illustrated by several studies (Figure 3). Protein-protein structural studies demonstrate that spike protein is the main driving force for host cell recognition (Ibrahim et al., 2020). A recent report demonstrated that spike protein had been O-glycosylated on the amino acid threonine (T678) adjacent to the furin cleavage site. Liquid chromatography-mass spectrometry analysis showed that the spike protein's LacdiNAc structural motifs and polyLacNAc structures (Sanda et al., 2021). The spike glycoprotein (S1) interacts with host cell epithelial angiotensin-converting enzyme 2 (ACE-2) receptors (ACE2). The nano-luciferase-based assay shows that the virus’s 1 protein has a strong binding affinity with the ACE-2 receptors (Lima et al., 2021). The ACE-2 is a transmembrane metallocarboxypeptidase, and it plays a significant role in the entry of the SARS-CoV-2 particle to the human lung epithelial cells. The ACE-2 degrades its substrate angiotensin II to angiotensin 1-7 and regulates RAS negatively, thereby protects the internal organs (Kuba et al., 2010). Reports illustrate the S-protein antigenic epitope of SARS-CoV-2 binds with the TLR4/MD-2 complex by strong molecular bonding interactions (Bhattacharya et al., 2020).

The human transmembrane protease serine 2 (TMPRSS2) processes the viral spike protein and exposes fusion peptide present in the S2 subunit to the host receptor ACE-2 (Qi et al., 2020). This S protein processing and priming by the TMPRSS2 is an essential step in the SARS-CoV-2 infection (Bestle et al., 2020). Then, the SARS-CoV-2 uses cysteine proteases like cathepsin B and L (CatB/L) and promotes virus-plasma membrane fusion (Zang et al., 2020). The pool of miRNA-based studies shows that TMPRSS2 acts as promising regulators for the SARS-CoV-2 entry checkpoint (Kaur et al., 2021). Further, it postulated that the SARS-CoV-2 downregulates the expression of ACE-2 resulted in the upregulated expression pattern of Ang II. Ang II is formed by the degradation of Ang I by the enzyme ACE-2 (D’ardes et al., 2020). This overexpressed Ang II binds with its plasma membrane receptor AT1R. This membrane-bound ATIR transduces the signals to the inflammatory transcription factors like NF-ƙB, which mediates several inflammatory cytokines' activation and overexpression (Mehta et al., 2020; Ye et al., 2020). Further, it has been recently reported that the AT1R phosphorylates JAK2 in the lung cells, which activates STAT-3 transduction to the nucleus (Seif et al., 2020). The STAT-3 is a signal transducer and activator of transcription, which initiates the active transcription of inflammatory cytokines. The release of extreme levels of proinflammatory cytokines like IL-1, IL-2, IL-6, IL-7, IL-10, and TNF-α during SARS-Co-2 infection has been named as “cytokine storm” (Merad and Martin, 2020). Additionally, the Ang II/AT1R interaction activates macrophages to produce excessive inflammatory cytokines and further contribute to “cytokine storm” and the development of Acute Respiratory Distress Syndrome (ARDS) (Sriram and Insel, 2020; Vellingiri et al., 2020). The cytokine storm led to multiple organ failure and subsequent mortality in severe COVID-19 infected patients (Henderson et al., 2020).

## COVID-19 and Renal Diseases

SARS-Co-V2 infection induces lung damage and respiratory failure and induces acute kidney failure and renal injury (Patel et al., 2020). Recent investigations confirmed that acute kidney injury has significantly caused mortality in the hospitalized COVID-19 patients (Zahid et al., 2020). The signal transduction pathway involved in acute kidney injury during COVID-19 has been linked with several factors (Benedetti et al., 2020). Cytokine storm and direct kidney cell infection through ACE-2 were proposed for acute kidney injury during COVID-19 infection (Ahmadian et al., 2020). The ACE-2 was expressed in proximal tubule epithelial cells, podocytes, glomerular endothelial cells, and kidney vasculature (Gheblawi et al., 2020; Magrone et al., 2020a). Reports illustrate that the renin/Ang/aldosterone system inhibitors during COVID-19 infection alter ACE2 expression and induced an increased mortality rate (Sriram et al., 2020; Vaduganathan et al., 2020). It has also been reported that the cytokine storm during COVID-19 infection accelerated the expression of a member of a family of apolipoproteins (APOL1), which resulted in podocyte damage, as severe acute tubular necrosis, as well as infiltration of macrophage and lymphocyte (Gupta et al., 2020; Kissling et al., 2020). Further, patients with functional defects in immunity were at an increased risk of chronic kidney disease during the SARS-CoV-2 infection (Cheng et al., 2020; Nadim et al., 2020).

## The Neurovirulence of SARS-CoV-2

Reports also illustrate that SARS-CoV-2 infection has also affected the central nervous system (CNS). Several neurological manifestations are associated with SARS-CoV-2 infection (Ellul et al., 2020). Acute encephalitis may be caused by the direct infection of brain tissue with the SARS-CoV-2 virus. COVID-19 infected patients showed the symptoms of olfactory and gustatory disturbances, headaches, dizziness, hallucinations, confusion, dysexecutive disorders, vigilance reduction, neuralgia, epileptic seizures, ataxia, sudden neurological deficits, and pyramidal tract sign (Lu Y. et al., 2020; Tsivgoulis et al., 2020; Lemprière, 2021). Immediately after infection, the COVID-19 affects the brain stem and cortex region through cerebrospinal fluid (CSF) (Sharifian-Dorche et al., 2020). Neurons and endothelial cells overexpress ACE-2 receptors (Verdecchia et al., 2020). The S1 proteins of SARS-CoV-2 bind with this receptor and induce neurological symptoms (Rhea et al., 2020; Xu and Lazartigues, 2020). It is thought that both direct respiratory failure and neurological damages are linked to the brain stem and cortex region damages (Fodoulian et al., 2020). Neuroinvasion of SARS-CoV-2 in the mouse brain has been reported (Song et al., 2021). Further, suitable experimental models are needed to reveal the potential complications of SARS-CoV-2 in neuroinflammatory disorders.

COVID-19 infection in the central nervous system (CNS) has attracted neurologists due to its neurological manifestations (Zhou and Kang, 2020). The COVID-19 viral particles have been found in the brain and cerebrospinal fluid (CSF) of the infected patients (Wu Y. et al., 2020). About 36% of COVID-19 patients develop neurological symptoms indicates that the virus acts as a neurotropic under certain pathological conditions. Reports show that COVID-19 infection causes encephalitis, encephalopathy, cerebrovascular pathologies, acute myelitis, and Guillain-Barré syndrome (Yachou et al., 2020). The K18-hACE2 mice infected with COVID-19 show anosmia with brain thrombosis (Zheng et al., 2021). The ACE-2 receptors were also found to be present in neurons which were responsible for neurotropism. Incubation of the BrainSpheres model with COVID-19 shows a higher fraction of viral particles infected neural cells (Bullen et al., 2020).

## Potential Molecular Targets of SARS-CoV-2 for Antiviral Therapeutics

Targeting the molecular pathways involved in the pathogenesis might provide a new avenue toward managing SARS-CoV-2 infection (Liu et al., 2021). Several molecular targets were explored to design and develop specific antiviral drugs for SARS-CoV-2 infection (Krishna et al., 2020). Computational and molecular docking studies explore spike S1 protein as a potential molecular target for developing effective therapeutic inhibitors (Wu C. et al., 2020). The S1 facilitates ACE2 mediated virus attachment, whereas the S2 subunit of spike protein facilitates membrane fusion (Shang et al., 2020). Wang Q. et al. (2020) illustrated the crystal structure of the C-terminal domain of spike (S) protein-bound with human ACE-2 (Wang Q. et al., 2020). Lan et al. (2020) also illustrated the receptor-binding domain’s crystal structure (RBD) of the spike protein complex with ACE-2. *In silico* docking experiments demonstrate the binding interaction of nelfinavir, an anti-HIV drug, with the spike proteins and illustrate S-n- and S-o-mediated membrane fusion (Musarrat et al., 2020). Molecular modeling and virtual screening analysis revealed that 14 natural compounds and 10 FDA-approved drugs with the highest binding energy (−8.1 kcal/mol) to the S-protein of SARS-CoV-2 (de Oliveira et al., 2020). Pandey et al. (2020) proposed dietary therapy and herbal medicine for COVID-19 prevention. Traditional medicines with antiviral and anti-inflammatory properties were also screened against spike proteins and ACE2 targets. Molecular dynamics simulation and docking studies revealed resveratrol and other stilbenoids as promising drug candidates against the viral protein-ACE2 receptor complex (Wahedi et al., 2020). Yu et al. (2020) screened 253 active Mangolian components using SARS CoV homology models. A pharmacoinformatics study illustrates the bioactive compounds from medicinal plants as a potential inhibitor of SARS-CoV-2 spike glycoprotein (Sinha et al., 2020). Chemical modification of phytochemicals has been reported to increase the potency and selectivity against SARS-CoV-2 infection. Experimental studies show that natural compounds like quercetin, caffeic acid, and myricetin act as inhibitors of SARS-CoV-2 infection (Mani et al., 2020; Mouffouk et al., 2021).

**FIGURE 4:**
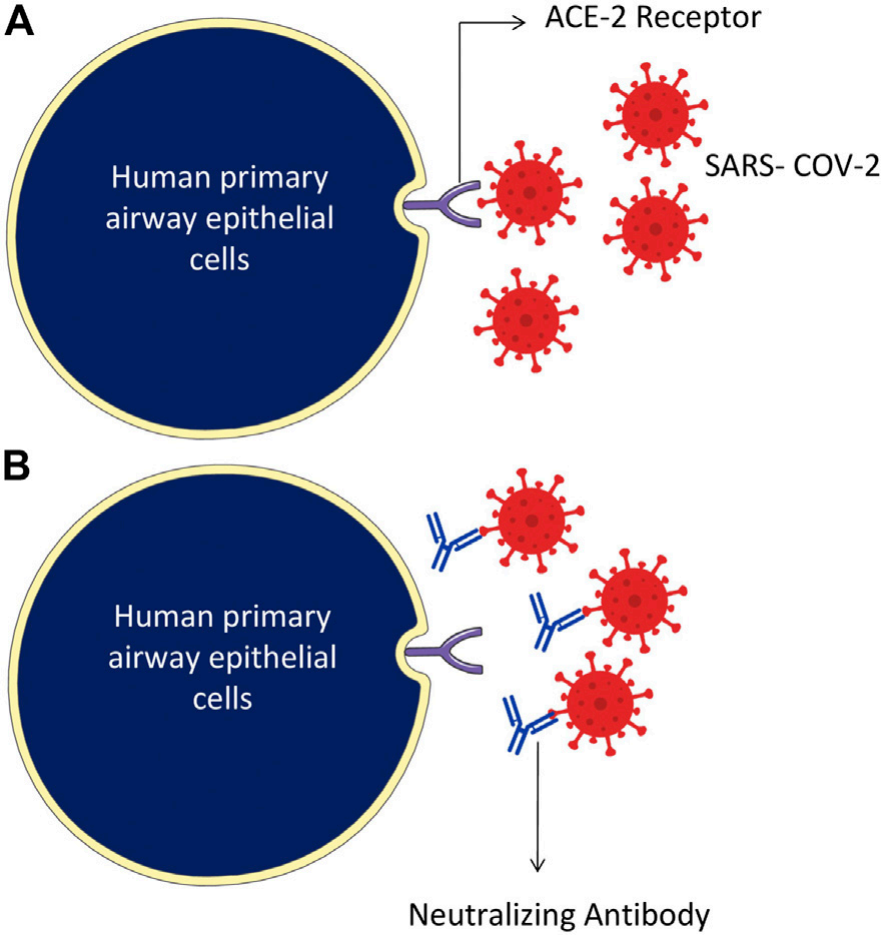
Humanized monoclonal antibodies neutralize the SARS-CoV-2 virus, specifically targeting by attaching to the RBD domain of spike protein on the surface of the virus. **(A)** The SARS-CoV-2 binds through RBD of the S protein and ACE-2 receptor of the host cells. **(B)** Humanized monoclonal antibodies bind with the virus spike proteins and neutralize them.

The SARS-CoV-2 binds through the RBD of the S protein and ACE-2 receptor of the host cells (Figure 4). Attempts have also been made to develop novel therapeutic recombinant ACE-2 antibodies, ACE inhibitors, AT1R blockers (Krishna et al., 2020). The role of recombinant human ACE-2 in ARDS therapy has already been proved (Zhang and Baker, 2017). Potent human IgG neutralization antibodies as clinical therapeutics candidates against SARS-CoV-2 Infection were developed (Wan et al., 2020). The applications of recombinant ACE-2-Ig in the treatment of SARS-CoV-2 infection have recently been demonstrated (Lei et al., 2020). Furthermore, several natural medicinal compounds act as inhibitors of ACE-2 (Junior et al., 2021). The natural luteoxanthin, violaxanthin, and rutin showed stronger binding efficiency with the ACE-2 receptor of SARS-CoV-2 (Upreti et al., 2021). Sulawesi propolis compounds have also been reported as ACE-2 inhibitors (Khayrani et al., 2021).

The transmembrane serine protease TMPRSS2 is essential for S protein priming to enter the SARS-CoV-2 virus into host cells. Therefore, inhibiting TMPRSS2 holds promise as an approach toward the treatment of SARS-CoV-2 infection. FDA-approved camostat mesylate (serine protease inhibitor) and bromhexine hydrochloride (BHH) serves as an inhibitor of TMPRSS2 proteolytic function (University of Aarhus, 2020). Another FDA-approved anti-inflammatory protein named alpha 1 antitrypsin (A1AT) has also effectively inhibited TMPRSS2 (Azouz et al., 2020). The bromhexine, a mucolytic drug used in treating respiratory disorders, has been found to inhibit TMPRSS2 selectively and prevent viral entry into the host cell (Depfenhart et al., 2020).

Besides, the crystal structure of SARS-CoV-2 main protease (Mpro) has recently been resolved (Jin and Du, 2020). The crystal structure of Mpro provides a basis for the design of potential inhibitors (Zhang L. et al., 2020). This molecular target was computationally screened with several FDA-approved antivirals (Hall and Ji, 2020). The Mpro of the SARS-CoV-2 plays a role in SARS-CoV-2 viral genome replication. Therefore, the Mpro protein serves as an attractive target for developing drug candidates against COVID-19. Jin and Zhao (2020) showed the structural basis of certain antineoplastic drugs to inhibit the Mpro enzyme. Durdagi et al. (2020) screened clinically approved drugs of NIH Chemical Genomics Center (NCGC) Pharmaceutical Collection (NPC) as potential inhibitors of Mpro and identified six promising drug candidates (Durdagi et al., 2020). Dai et al. (2020) illustrated the X-ray crystal structures of Mpro in complex with the structure-based designed compounds 11a or 11b (1.5 Å). In animal models, both the compounds exhibited good PK properties with low toxicity (Dai et al., 2020). Jin and Zhao (2020) showed the X-ray crystal structure of Mpro in complex with carmofur with very low EC_50_ values. The combinatorial design of peptide-based inhibitors was developed by targeting the dimerization of Mpro (Goyal and Goyal, 2020). FDA-approved antiplatelet cilostazol also effectively inhibits Mpro of SARS-CoV-2 (Abosheasha and El-Gowily, 2020). Enmozhi et al. (2020) showed andrographolide, a plant terpenoid, as an inhibitor of Mpro through *silico* studies. Gentile et al. (2020) screened marine natural products as inhibitors of SARS-CoV-2 main protease.

The RNA-dependent RNA polymerase [(RdRp), also named nsp12] of SARS-CoV-2 is the central component of viral replication. It is considered to be a primary target for the development of antiviral therapeutics. Recently, Gao Y. et al. (2020) reported full-length nsp12 in complex with cofactors nsp7 and nsp8 by electron microscopy (2.9 Å). Researchers repurposed anti-HCV nucleotide inhibitors against SARS-CoV-2 RdRp (Elfiky, 2020a). The cryo-EM structure of RdRp (2.8 Å) with replicating RNA and remdesivir was recently revealed. This study indicates that remdesivir mimics like an RNA nucleotide and covalently linked elongating RNA, thereby inhibits further replication of viral RNA molecule (Yin et al., 2020). The effectiveness of FDA-approved antiviral drugs like ribavirin, remdesivir, sofosbuvir, galidesivir, and tenofovir were tightly linked with RdRp. The other agents like guanosine derivative (IDX-184), YAK, and setrobuvir were top antiviral drugs with very high specificity to SARS-CoV-2 RdRp (Elfiky, 2020b; Elfiky, 2020c). Du and Chen (2020) showed the pharmacokinetics of favipiravir, a potent RdRp inhibitor approved for use in influenza, and demanded to design clinical trials for favipiravir against COVID-19 (Thomas et al., 2020). Lung et al. (2020) showed theaflavin could be a potential SARS-CoV-2 RdRp inhibitor.

Reports illustrate that respiratory failure during COVID-19 pathogenesis has generally been associated with the activation of inflammatory transcription factors (Yarmohammadi et al., 2021). In response to the activation of transcription factors the inflammatory markers like interleukin 6, interleukin 8, VEGF, MCP-1, and E-cadherin have been overexpressed (Abers et al., 2021). Researchers also target the transcription factors involved in SARS-CoV-2 pathogenesis. Telmisartan effectively downregulates AT1R by acting as an agonist of Peroxisome Proliferator-Activated Receptor-gamma (PPAR-gamma). JAK-STAT-2 signaling inhibition has also been proposed as a new treatment strategy for patients with SARS-CoV-2 infection (Seif et al., 2020). *Cannabis sativa* extracts can down-regulate the expression of the two critical receptors for SARS-CoV-2 in several human epithelial models *via* PPAR-gamma modulation (Esposito et al., 2020). It is proposed that treatment with glucocorticoids, AT1R inhibitor, and retinoic acids might modulate NF-κB signaling, reducing the “cytokine storm” (Banu et al., 2020; Li Z. et al., 2020; Magrone et al., 2020b). Tocilizumab has also interacted with mIL-6R and sIL-6R and subsequently inhibits JAK-STAT and MAPK/NF-κB-IL-6 signaling pathways (Saha et al., 2020b). Horowitz and Freeman (2020) hypothesized and recommended a randomized controlled trial by modulating NF-κB and subsequent cytokine formation to manage SARS-Cov-2 complications. An integrative pathway network analysis study illustrates that SARS-CoV-2 miRNAs target NF-KB, JAK/STAT3, TGF beta signaling transduction pathways, and cellular epigenetic regulation pathways (Aydemir et al., 2021).

## Experimental Models for the Study of SARS-CoV-2 Pathogenesis

### Cellular Models

**FIGURE 5:**
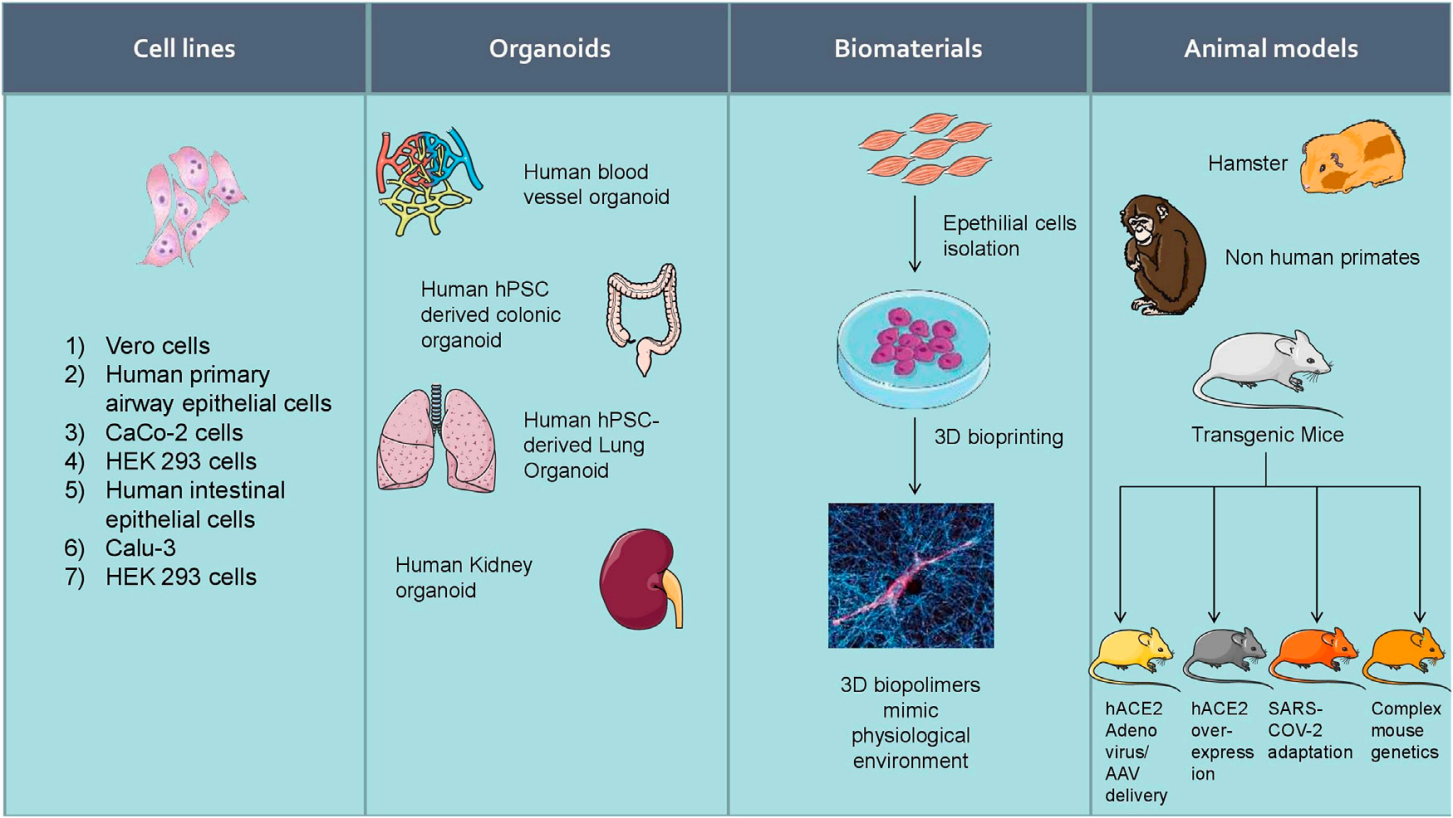
Experimental models to study the pathogenesis of SARS-CoV-2 infection and study drug candidates’ pharmacological action.

Increasing experimental evidence illustrate several potential molecular targets for the treatment of SARS-CoV-2 infection. As the SARS-CoV-2 pandemic is alarmingly progressing, there is an urgent need to develop reliable cellular and animal models to understand the mechanism of pathogenesis and to apply this knowledge to develop therapeutic countermeasures. The development of clinically relevant experimental models is essential to examine the pathogenesis of COVID-19 in different organs (Chugh et al., 2021). Researchers started using several experimental models to study the pathogenesis of SARS-CoV-2 infection and study drug candidates' pharmacological action (Figure 5). Further, the cellular models were used to analyze viral titer values of infectious samples isolated from the patients. Moreover, the cellular models were used to overexpress specific SARS-CoV-2 proteins to analyze 3 D crystal structure of proteins (Table 2).

**TABLE 2:** List of cell lines and organoids used for SARS-CoV-2 culture, treatment, and prevention strategies.

Experimental model	Pharmacological studies	References
**Eukaryotic cellular models**		
Human primary airway epithelial cells	An orally bioavailable *β*-D-N4-hydroxycytidine (NHC; EIDD-1931) and its derivatives were tested to inhibit SARS-CoV-2 infection using human primary airway epithelial cells	Sheahan et al. (2020)
	Single-cell RNA sequencing was conducted to understand transmembrane receptors’ expression pattern to bind the SARS-CoV-2 virus	Lukassen et al. (2020)
	The entry of SARS-cov-2 in polarized Calu-3 lung epithelial cells was illustrated	Tseng et al. (2005)
	Compared to the expression pattern of ACE-2 and TMPRSS2 in primary lung epithelial cell controls	Abo et al. (2020)
	Studied the expression levels of the ACE2 receptor to understand the binding interaction of SARS-CoV-2 in the human airway epithelium	Zhang H. et al. (2020)
	Studied the potential of remdesivir to inhibit SARS-CoV-2 in human primary lung cells	Pruijssers et al. (2020)
	The human reconstituted airway epithelial model was tested for remdesivir therapeutic efficacy	Pizzorno et al. (2020)
Vero cells	Studied the potential of ivermectin as an inhibitor of SARS-cov-2 in Vero-hSLAM cells	Caly et al. (2020)
	Studied the efficacy of IFN-α or IFN-β against SARS-cov-2 viral titers in vero cells	Mantlo et al. (2020)
	Observed anti-ACE2 against viral replication in vero E6 cells	Hospital Universitario Ramón y Cajal (2003)
	Identified the SARS-cov-2 virus replication in Vero-CCL81 and vero E6 cells	(Harcourt et al., 2020)
	Observed the localization of CD147 in SARS-cov-2 affected vero E6 cells	Wang K. et al. (2020)
	Studied the potential of nelfinavir as an active therapeutic agent against COVID-19 in vero E6 cells	Xu et al. (2020)
	Employed VeroE6 cells for virus isolation and culture	Hui et al. (2020)
	The potential of lianhuaqingwen against SARS-cov-2 infection was observed using the cytopathic effect (CPE) and plaque reduction assay in vero E6 cells	Runfeng et al. (2020)
	Vero-6 cells were infected for the titration of infectious SARS-cov-2 particles by plaque-forming assays	Mendoza et al. (2020)
	The inhibitory effect of liu shen capsule against SARS-cov-2 replication was evaluated by CPE and plaque reduction assay in vero E6 cells	Ma et al. (2020)
CaCo-2 cells	Employed CaCo-2 cells for culturing COVID-19 obtained from air and environmental samples	Zhou and Otter (2020)
	CaCo-2 cells were used for SARS-cov-2 isolation from clinical specimens	Kim et al. (2020)
	SARS-CoV-2 viral RNA present in the infected cardiomyocytes induced productive infections in CaCo-2 cell lines	Bojkova et al. (2020)
	Assessed viral replication and proinflammatory responses to human macrophages and Caco-2 cells	Hui et al. (2020)
CaCo-2 cells	Analyzed gene expression pattern of SARS-cov-2 infections using single-cell transcriptomics in H1299, Caco-2, and Calu-3 cells	Emanuel et al. (2020)
H1299		
Calu-3		
Calu-3	A clinically proven protease inhibitor, camostat mesylate, inhibits Calu-3 infection caused by SARS-cov-2	Huang et al. (2020)
HEK 293 cells	The flow cytometric approach employed to assess spike-specific IgG and IgM antibody responses	Lapuente et al. (2020)
	Mapped the expression pattern of N-glycosylation on hACE2 on human HEK 293 cells	Shajahan et al. (2020)
	Full-length human ACE2 was expressed HEK 293 F cells, purified and used for the structural determination of ACE2	Yan et al. (2020)
Human intestinal epithelial cells	Human intestinal epithelial cells used for the production of SARS-CoV-2 virus particles	Stanifer et al. (2020)
**Organoids**		
Human derived blood vessel organoids	Tested the efficacy of hrsACE2 on SARS-CoV-2 infected organoids	Monteil et al. (2020)
Human kidney organoids		
Human iPSC-3D organoids	Used as a potential *ex vivo* infection model for novel treatment and prevention strategies	Zhou and Liu (2020)
hPSC-derived lung organoids	Analyzed transcriptome analysis after SARS-CoV-2 infection	Han et al. (2020)
	Performed a high throughput and identified FDA-approved as inhibitors of SARS-cov-2 entry	
Human hPSC derived colonic organoids (hPSC-COs)	Conducted single-cell RNA-seq and immunostaining to show entry of viral particles through ACE-2; hPSC-COs organoids were employed as a high-throughput screening system for FDA-approved drugs	Duan et al. (2020)
Human induced pluripotent stem cell (iPSC)- derived BrainSphere model	Allow both COVID-19 infection and serves as an experimental model for nerotropism of COVID-19	Bullen et al. (2020)

Human lung epithelial cellular models are the most prominent platform for SARS-CoV-2 infectious studies (Sungnak et al., 2020). Lung-specific extracellular matrix 3D cell cultures have been introduced by scientific companies that enable them to generate more relevant scientific data (Li Y. et al., 2020). Apart from the alveolar cellular models, the ACE-2 expression has been reported in other organs such as kidney and gut-derived cellular models. Hoffmann et al. showed that treating Vero-E6 cells, a monkey kidney cell line, with an Anti-ACE-2 Antibody, blocked VSV pseudotypes’ entry expressing the S protein (Hoffmann et al., 2020). The cytokine storm occurring during SARS-CoV-2 infection can effectively be analyzed using human PBMC cellular models (Thomas et al., 2020).

Indeed, the experiments with SARS-CoV-2 should be conducted at biosafety level 3. There is a clear challenge in the laboratory diagnosis and cultivation of this deadly SARS-CoV-2 viral particle. Scientific methods and guidelines have to be followed to enable safety while handling RNA, DNA, and proteins from the SARS-CoV-2 infected cells (Jureka et al., 2020) illustrated methods to culture SARS-CoV-2 in multiple cell lines like Vero E6, Calu-3, CaCo-2, Huh7, A549, and 293T cell lines and measured virus infectivity by agarose SARS-CoV-2 plaque assay. Recent investigations revealed that viral infectivity depends on the presence of ACE-2 and TMPRSS2 (Hoffmann et al., 2020). Therefore, the researchers overexpress the SARS-CoV-2 entry receptor ACE-2 or the Spike processing cellular protease TMPRSS2 in the experimental cell lines (Matsuyama et al., 2020).

### Human Pluripotent Stem Cell-Based Platform

There is an urgent need for the development of relevant organoid type physiological models to study SARS-CoV-2 infection. The hPSC-derived cells/organoids provide valuable models for understanding human tissues’ cellular responses to SARS-CoV-2 infection and disease modeling of COVID-19 (Han et al., 2021). Recent clinical studies show a strong association between COVID-19 and diabetes (Muniyappa and Gubbi, 2020). The ACE-2 protein was expressed in the islet and exocrine tissue microvasculature and a subset of pancreatic ducts. Results show that human pancreatic beta cells and liver organoids were permissive to COVID-19 infection (Coate et al., 2020). Adult primary human islets, hepatocyte, and cholangiocyte organoids have also served as COVID-19 experiment models (Zhou et al., 2020; Lamers et al., 2021).

The hPSC-derived cells and organoids serve as a platform for SARS-CoV-2 tissue tropism. Further, human pancreatic alpha and beta cells were also used to study SARS-CoV-2 infection (Yang et al., 2020). It has also been found that human hepatocyte and cholangiocyte organoids show permissive conditions for the culture of SARS-CoV-2 (Chandar et al., 2020). It has been found that hPSC-derived cells/organoids show similar chemokine responses as occurs in COVID-19 tissues (Zhou et al., 2020); (Salahudeen et al., 2020).

The COVID-19 infects human neuronal progenitor cells and experimental 3D brain organoids (Zhang B. Z. et al., 2020). Thus, human-induced pluripotent stem cell (iPSC)- derived BrainSphere serves as a reliable state-of-the-art 3D organotypic cell culture model for COVID-19 infection. Incubation of the BrainSpheres model with COVID-19 shows a higher fraction of viral particles infected neural cells (Bullen et al., 2020). This lab-grown BrainSphere model has been used for neurotoxicity studies of COVID-19 in a simple BSL-3 environment. Further, researchers illustrated COVID-19 infects two different iPSC-derived (IMR90 and Crx-iPS) human 3D cerebral organoids and causes Tau abnormalities and neuronal cell death (Ramani et al., 2020). Further, COVID-19 infects choroid plexus of brain and alters the CSF-blood brain barrier in experimental human organoids (Pellegrini et al., 2020). Additionally, the neuroinvasive and neurodegeneration potential of SARS-CoV-2 has also been revealed in 3D human brain organoid models (Song et al., 2020).

Liver organoids were also to be infected by COVID-19. The COVID-19 infection induces tissue damage in human liver ductal organoids *ex vivo*, and the liver organoids serve as a model for the studies of tropism and pathogenesis of SARS-CoV-2 (Zhao B. et al., 2020). The intrahepatic bile duct cells grown using a human liver organoid platform have effectively been infected by COVID-19 and illustrate the mechanism for SARS-CoV-2 liver injury (Chandar et al., 2020).

### Biomaterials Based Models

The organomimetic 3D bioprinting technology mimics the physiological environment for the study of SARS-CoV-2 infection. The 3D bioprinted lung-like structures act as an air–tissue interface with open architecture and multiple cell types. These 3D lungs, bronchiolar, or alveolar models have been prepared for the studies of SARS-CoV-2 infection (Chakraborty et al., 2020). Recently, the pathological behavior of SARS-CoV-2 and the efficacy of therapeutic agents were analyzed by using hydrogel-based high-precision 3D bioinks (Choi et al., 2021). Several biomaterials display distinct structural characteristics for COVID-19 related research (Jarai et al., 2021).

### Transgenic Mice Models

Animal studies are essential to understand the mode of action, absorption, mode of administration, pharmacokinetics, and pharmacodynamics of the drugs that inhibit SARS-CoV-2 molecular targets (Pandey et al., 2020). Animal models are well suited for the invention of potential vaccines or antivirals. Currently, there is no specific reported animal model to study the pathogenesis of SARS-CoV-2 and the treatment outcome of therapeutic agents (Rockx et al., 2020). However, several investigations are currently undergoing animal models such as macaques, cats, ferrets, hamsters, and transgenic mice ACE-2 (hACE-2) (Cleary et al., 2020). The Jackson Laboratory, United States of America is currently producing different transgenic mouse models suitable for SARS-CoV-2 infection (Callaway, 2020). A vector carrying a human ACE-2 sequence has been introduced to the genome of wild-type mice regulated by the human cytokeratin 18 (K18) promoter in mouse epithelial cells. The SARS-CoV-2 causes fatal infection in transgenic K18-hACE2 mice *via* hACE2 receptors (Cleary et al., 2020; Lutz et al., 2020). The experimental mice transduced with Ad5-hACE2 develop viral pneumonia with a widespread infection of the lungs (Borges et al., 2020).

The laboratory wild-type mice will not be a suitable model for antiviral therapeutics development. The SARS-CoV-2 viral particle induces interstitial pneumonia and macrophages infiltration in the human lung tissue. The bronchial and alveolar epithelial cells are the primary targets of SARS-CoV-2 infection. However, it has been found that low interactions between the viral S protein and the mouse ortholog of the human ACE-2 receptor (Dinnon et al., 2020). Therefore, the researchers generate a transgenic mouse that overexpresses human ACE-2 by “knocked-in” methods and sensitizes the laboratory mouse for SARS-CoV-2 infection. Jiang et al. recently developed SARS-CoV-2 hACE-2 transgenic C3B6 mice. This infected mice model shows typical interstitial pneumonia and pathology similar to SARS-CoV-2 infected patients (Jiang et al., 2020). Recent experimental evidence indicates that the SARS-CoV-2 pathogenicity was higher in transgenic hACE-2 mice than the wild-type laboratory mice (Bao et al., 2020). The pathogenicity of SARS-CoV-2 in hACE-2 mice fulfilled Koch’s postulates and the transgenic mouse model may facilitate the development of therapeutics and vaccines against SARS-CoV-2 (Bao et al., 2020). Transgenic mice that express hACE2 in the epithelial cells can develop a lethal SARS-CoV-2 infection after intranasal inoculation (Lutz et al., 2020). Dinnon et al. (2020) produced a mouse model that supports the study of IFN lambda-1a treatment as similar to human COVID-19 infection. Israelow et al. (2020) reported developing a mouse model of SARS-CoV-2 based on adeno-associated virus (AAV)-mediated expression of hACE-2. These mice support viral replication and exhibit pathologic findings found in COVID-19 patients, and these mice were also used for antibody production. Another animal study illustrated the inhibitory role of angiotensin receptor blockers (ARBs) against SARS-CoV2 mediated pneumonia (Kai and Kai, 2020). The laboratory mice transduced with human ACE2 developed pneumonia after COVID-19 infection and neutralized by mAbs with an attenuated lung infection and inflammation (Hassan et al., 2020). A recent study shows the fatal neuroinvasion of SARS-CoV-2 in transgenic K-18-hACE2 mice (Carossino et al., 2021). Another study also illustrates infection of CNS cells and encephalitis by SARS-CoV-2 with the inflammatory response in K18-hACE2 mice (Kumari et al., 2021). K18-hACE2 transgenic mice pre-treated with convalescent plasma prevented most signs of severe pneumonia. Thus, K18-hACE2 mice showed molecular pathogenesis of COPVID-19 infection and suitable for therapeutic intervention studies (Zheng et al., 2021). The adeno-associated virus that expresses human ACE2, either Ad5-hACE2 or AAV-hACE2, develops an infection in the mouse lungs like viral pneumonia. Therefore, mice sensitized with Ad5-hACE2 or AAV-hACE2 might be helpful in the studies of testing vaccines and antiviral therapeutics (Muñoz-Fontela et al., 2020).

### Non-Human-Primate Models

Nonhuman primate models were suitable for the development of therapeutics and vaccines for COVID-19 to clinical trials. Pathology and pathogenesis of SARS-CoV-2 infection were compared in the lungs of different nonhuman primates like rhesus macaques, baboons, and marmosets (Singh et al., 2021). Effective treatment of SARS-CoV-2-infected rhesus macaques by attenuating inflammation (Lu et al., 2021). Researchers illustrate that the intranasal vaccination of ChAdOx1 nCoV-19/AZD1222 reduces COVID-19 infection and prevents the shedding of SARS-CoV-2 D614G compared to intramuscular vaccination using rhesus macaques as an experimental model (van Doremalen et al., 2021). The immunogenicity and protective efficacy of vaccine candidates (mRNA-1273, followed by Ad26.CoV2.S, NVX-CoV2373, BNT162b2, RBD, and BBV152) were tested in preclinical nonhuman primate models, and the results were correlated with the clinical trial data (Mukhopadhyay et al., 2020). Preclinical trials of COVID-19 vaccine candidates in NHPs yielded promising results, with some candidates faring better than others. The immunogenicity and protective efficacy of a single dose of adenovirus serotype 26 (Ad26) vector-based vaccines are expressing the SARS-CoV-2 spike (S) protein in nonhuman primates (Mercado et al., 2020). Baricitinib is a clinically approved JAK inhibitor that exhibits a therapeutic effect against SARS-CoV-2 infection in the rhesus macaque model. Baricitinib-treated animals showed suppression of cytokines and chemokines production in nonhuman primates (Hoang et al., 2021). However, standardized protocols are still needed to compare vaccine efficacy in nonhuman primates.

## Conclusion

The pandemic outbreak of SARS-CoV-2 infection has created a severe health problem worldwide as it causes severe ARDS. Conversely, no vaccine and specific drugs are available for the treatment of SARS-CoV-2 infection. Present clinical treatment regimes are inadequate to overcome the viral replication in the human host cells and prevent organ failure. Therefore, there was a growing research interest among the researchers to understand the biology of SARS-CoV-2. Understanding the viral druggable molecular targets helps us design structure-based inhibitors for effective antiviral therapy and develop vaccination strategies. Besides cellular and animal models, the human and animal organoids currently play a significant role as an experimental SARS-CoV-2 platform. The present review illustrates fundamental research and clinical trials using *in vitro* cell lines, human organoids, and transgenic ACE-2 mice as experimental models.

Further, this review explored the potential druggable molecular targets to study the therapeutic agents for their efficacy. We believe that the present review may guide the basic researchers to select suitable experimental models for their pharmacological and clinical studies. This will meet the clinicians to design better treatment strategies for the ongoing SARS-CoV-2 pandemic.
